# Translational utility of a hierarchical classification strategy in biomolecular data analytics

**DOI:** 10.1038/s41598-017-14092-7

**Published:** 2017-11-03

**Authors:** Dieter Galea, Paolo Inglese, Lidia Cammack, Nicole Strittmatter, Monica Rebec, Reza Mirnezami, Ivan Laponogov, James Kinross, Jeremy Nicholson, Zoltan Takats, Kirill A. Veselkov

**Affiliations:** Computational and Systems Medicine, Department of Surgery and Cancer, Faculty of Medicine, Imperial College London, London, United Kingdom

## Abstract

Hierarchical classification (HC) stratifies and classifies data from broad classes into more specific classes. Unlike commonly used data classification strategies, this enables the probabilistic prediction of unknown classes at different levels, minimizing the burden of incomplete databases. Despite these advantages, its translational application in biomedical sciences has been limited. We describe and demonstrate the implementation of a HC approach for “omics-driven” classification of 15 bacterial species at various taxonomic levels achieving 90–100% accuracy, and 9 cancer types into morphological types and 35 subtypes with 99% and 76% accuracy, respectively. Unknown bacterial species were probabilistically assigned with 100% accuracy to their respective genus or family using mass spectra (n = 284). Cancer types were predicted by mRNA data (n = 1960) for most subtypes with 95–100% accuracy. This has high relevance in clinical practice where complete datasets are difficult to compile with the continuous evolution of diseases and emergence of new strains, yet prediction of unknown classes, such as bacterial species, at upper hierarchy levels may be sufficient to initiate antimicrobial therapy. The algorithms presented here can be directly translated into clinical-use with any quantitative data, and have broad application potential, from unlabeled sample identification, to hierarchical feature selection, and discovery of new taxonomic variants.

## Introduction

The relentless drives towards precision medicine necessitate an ever-increasing reliance on integrating of complex and large-scale multi-omics and clinical datasets derived from multiple sources^1^. In the real world, these sets often have complex and diverse data structures covering non-linear relationships and often missing data points. In the case of microbiome research, there is a considerable need to integrate metagenomic information and other systems parameters such as metabolism. In most cases, the primary requirement is to predict multiple classifications and outcomes based on multivariate data and modeling such as the use of hierarchical classification (HC) strategies. Although HC methods have been used extensively in research applications such as text classification^2–4^, and limitedly in genomics^5^ and genome sequencing^6^, the translation into clinical science and practice remains limited. Here we present a generalized HC implementation in the biomedical context, demonstrate its performance on biomedical datasets, and discuss its immediate translational advantages and uses.

Current commonly used bioinformatics methods for classification of biomedical datasets are based on ‘training’ a single classification method (or ensemble, thereof) to discriminate between different classes e.g. patient outcomes or healthy and cancerous tissue^7^, or organisms such as bacterial species^8,9^. Such approaches may be referred to as ‘flat classification’ and while this provides classification accuracy results which seem highly promising, there are key limitations which are often overlooked: (i) class discrimination at one level may diminish with increasing numbers of classes, resulting in lower classification accuracy for large datasets; (ii) since all classes in the model are considered to be either ‘training’ or ‘prediction’, the classification accuracy for a particular class can be influenced by other taxonomically ‘distant’ classes; and (iii) incomplete databases offer little or no predictive capacity for new (previously unknown) classes. In the microbiology field, for example, the continuous discovery of new species and the emergence of new bacterial strains means that a ‘complete’ database is impossible to compile. The same is true, though to a lesser extent, for undifferentiated cancers, where identification of cancer type is not possible in certain instances, limiting targeted therapeutic intervention.

To mitigate these limitations, here we propose a hierarchical ‘top-down’ classification algorithm, where classes are taxonomically classified and predicted. Unlike ‘flat classification’, a hierarchical approach enables the training of a number of models, one for each classification problem, considering hierarchical relationships. This approach subdivides classification into smaller and simpler classification problems^2^ and may in turn result in improved classification accuracy since classes unrelated taxonomically to a given class of interest are not considered in the classification of the latter. The advantage of HC is further amplified in circumstances where the quality of data for some classes may not be comparable to others. While in ‘flat classification’ the inferior data quality of some classes may potentially affect all others, in hierarchical classification, taxonomically-distinct classes are less susceptible to this phenomenon.

One of the most significant advantages of adopting a HC approach that has not been realized or utilized before, particularly from a clinical application point of view, is that it provides the ability to predict a class at different classification tiers. While the specific class for classes not contained within the existing database may not be retrieved, this enables the prediction of upper tier classes. This information may still be highly clinically relevant as in many cases it would be adequate to determine appropriate treatment. For example, in the case of treatment of bacterial infections, where the specific species is not always identifiable.

Based on their cell wall structure, bacteria are classified as either Gram-positive or Gram-negative and, similar to other living organisms, taxonomically hierarchically classified down to the species level, thus prediction of upper hierarchy class is often sufficient for the appropriate antibiotic regimen to be prescribed. The identification of bacteria using mass spectra has been reported and reviewed several times^9–15^, however prediction is difficult to achieve with the use of conventional classification methods which are frequently incapable of correctly assigning upper level taxonomy for species not encountered before in the dataset.

Here we apply and validate a HC and prediction approach to large-scale clinically relevant spectroscopic and genomic datasets; we demonstrate accurate prediction of bacterial species and cancer subtypes using mass spectral and gene expression profile data, respectively. A broad range of potential translational applications beyond those used to validate the algorithm are also outlined.

## Results

### Classification performance

**Figure 1:**
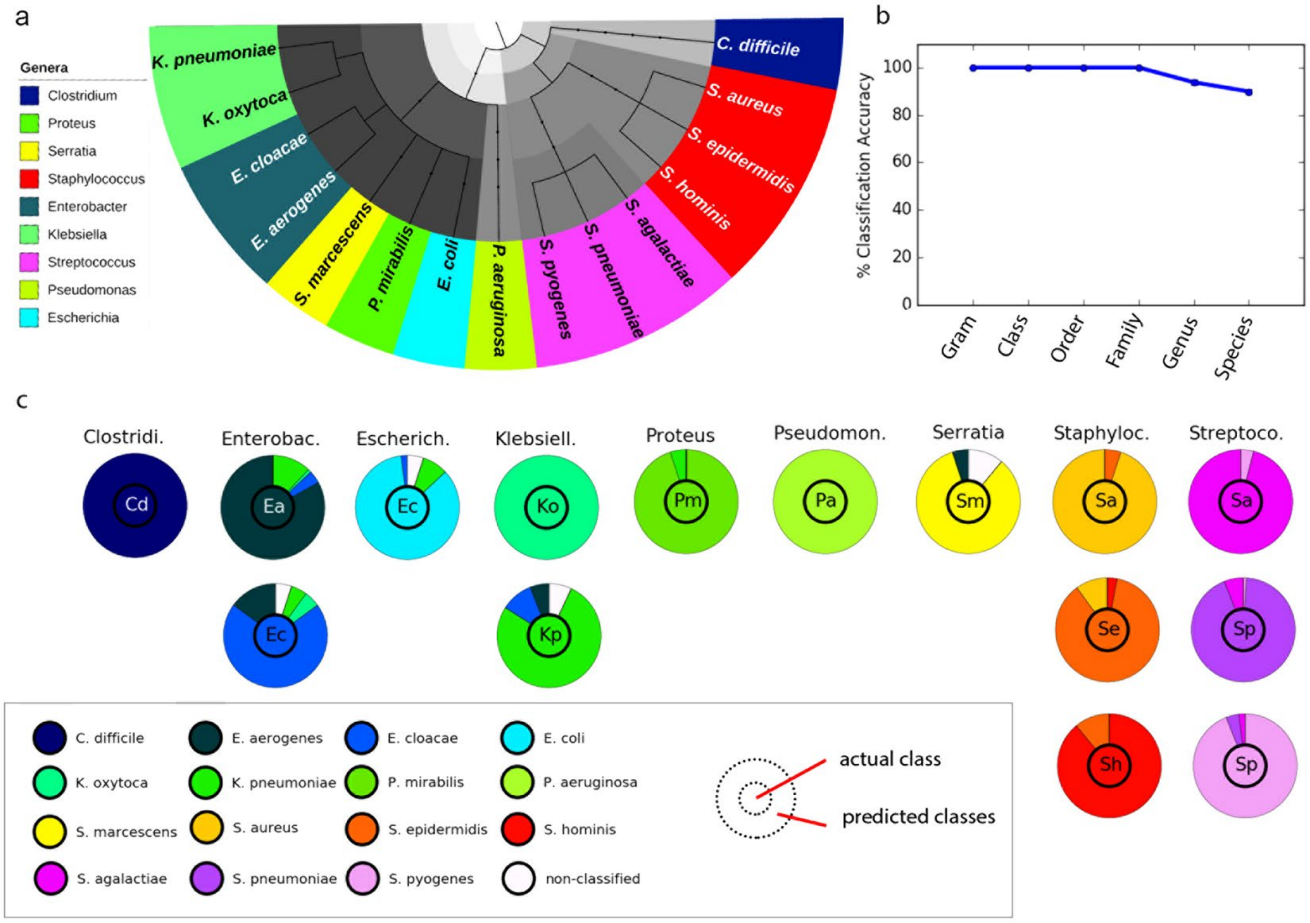
Hierarchical classification of bacterial mass spectral profiles. (**a**) Hierarchical tree structure for the bacterial species analyzed, where color-coding represents species belonging to the same genus, as indicated in the legend. Grey-scaling indicates upper level hierarchies; (**b**) Plot of the mean % classification accuracies for 5 predictions at the different tree levels achieved by the selective classifier approach; (**c**) Semi-quantitative plot showing the classification performance at the lower-most/species level, as well as where misclassifications occurred. The inner circle indicates the actual species while the outer circle indicates the predicted class. Each column represents a genus while rows represent one or multiple species belonging to the respective genus. The overall color for the species in each genus corresponds to the color legend in (**a**).

To validate and assess the performance of the proposed HC algorithm, we first applied it to mass spectral profiles acquired from 15 bacterial species (Fig. 1a), determining the classification performance across 6 hierarchy levels: (i) Gram staining type; (ii) class; (iii) order; (iv) family; (v) genus; and (vi) species. An average classification accuracy of 100% at the top 4 levels was achieved, with 94 ± 1% and 90 ± 1% at the genus and species levels, respectively (Fig. 1b). At the species level (Fig. 1c; Supplementary Table S1), *Clostridium difficile*, *Pseudomonas aeruginosa* and *Klebsiella oxytoca* were correctly classified with 100% accuracy. *Staphylococcus* spp. and *Streptococcus* spp. were misclassified into same-genus species.

Species belonging to the *Enterobacteriaceae* family (level 4; specifically: *Enterobacter* spp., *Escherichia* spp., *Klebsiella pneumoniae*, *Proteus mirabilis* and *Serratia marcescens*) were misclassified into same-family species. *Enterobacter* spp. were misclassified between each other as well as into *Klebsiella* spp., and vice-versa *Klebsiella pneumoniae* was misclassified into *Enterobacter* spp. *Serratia marcescens* was misclassified into *Enterobacter aerogenes* and *Proteus mirabilis* was misclassified into *Klebsiella pneumoniae*. 5–11% of *Enterobacter cloacae*, *Escherichia coli*, *Klebsiella pneumoniae* and *Serratia marcescens* were not classified. Score plots for a selection of models are presented in Supplementary Fig. S2. PLS was the most chosen method across the hierarchical tree. The different methods demonstrated equivalent predictive capacity at upper hierarchical levels and therefore PLS was chosen since it is the most computationally-efficient method.

**Figure 2:**
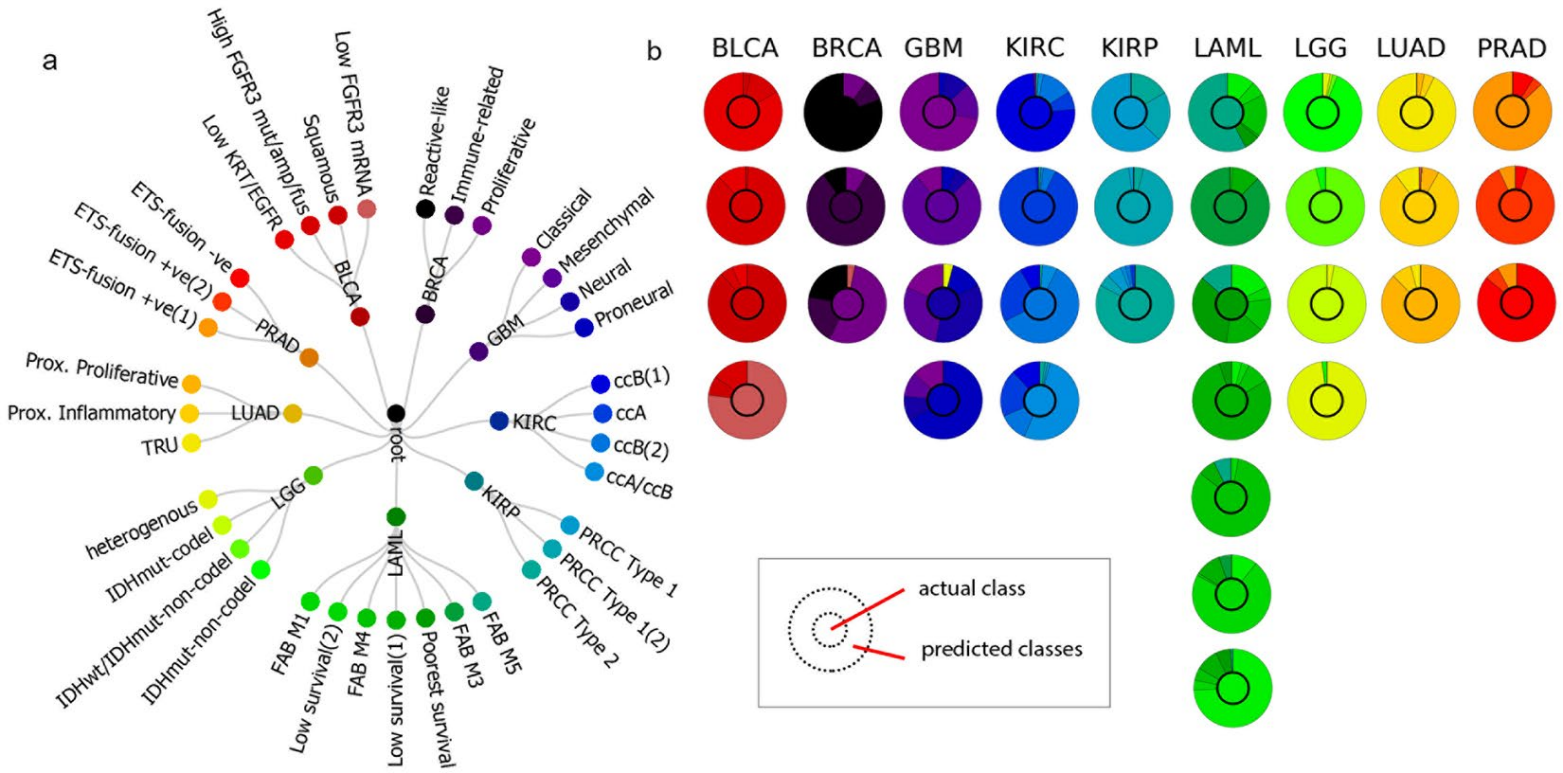
Cancer genomic dataset hierarchical classification. (**a**) Hierarchical tree structure for the cancer dataset analyzed derived from previous literature, where cancer types (level 1) were classified with a mean accuracy of 99% while subtypes (level 2) were classified with a mean accuracy of 76 ± 2%; (**a**) Semi-quantitative plot showing the classification performance at the bottom-most level/cancer sub-type level, as well as where misclassifications occurred. The inner circle indicates the actual class while outer circle indicates the predicted class. Columns represent the different cancer types while rows represent corresponding sub-types. Sub-type colors correspond with the node colors assigned in the lower-most layer of the hierarchical tree (**a**).

We assessed the approach further by applying it to a large publicly-available cancer gene expression dataset. We defined a two-level hierarchy: (i) morphological cancer type (level 1); and (ii) molecular cancer sub-type (level 2), derived from previous literature (Fig. 2a). Nine cancer types were classified with an overall accuracy of 99% at the first hierarchy level, with classification accuracies ranging from 98–100%. Kidney renal clear cell carcinoma (KIRC) and kidney renal papillary cell carcinoma (KIRP) were classified with 98% accuracy (misclassifications occurring between the same cancer types), breast adenocarcinoma (BRCA), glioblastoma multiforme (GBM), and lung adenocarcinoma (LUAD) were predicted with 99% accuracy with <1% misclassified into bladder urothelial carcinoma (BLCA) and lower grade glioma (LGG). Acute myeloid leukemia (LAML), LGG, and BLCA were correctly classified in all cases.

Average percentage accuracies were consistent across multiple cross-validation repetitions. Overall classification of the 35 cancer subtypes (level 2) was achieved with a mean accuracy of 76 ± 2%, with individual subtype classifications ranging widely from 35–97%. Lowest classification was recorded for a LAML subtype (‘poorest survival’ subtype), 19 out of 35 were classified with over 80% accuracy, 10 were classified with 60–80% accuracy, and 6 subtypes were classified with less than 60% accuracy. With the high classification accuracies at the first level, misclassification of cancer subtypes mostly occurred between subtypes of the same cancer. Subtype misclassifications are shown semi-quantitatively in Fig. 2b. Quantitative accuracies for all subtypes are listed in Supplementary Table S2.

### Prediction performance

**Figure 3:**
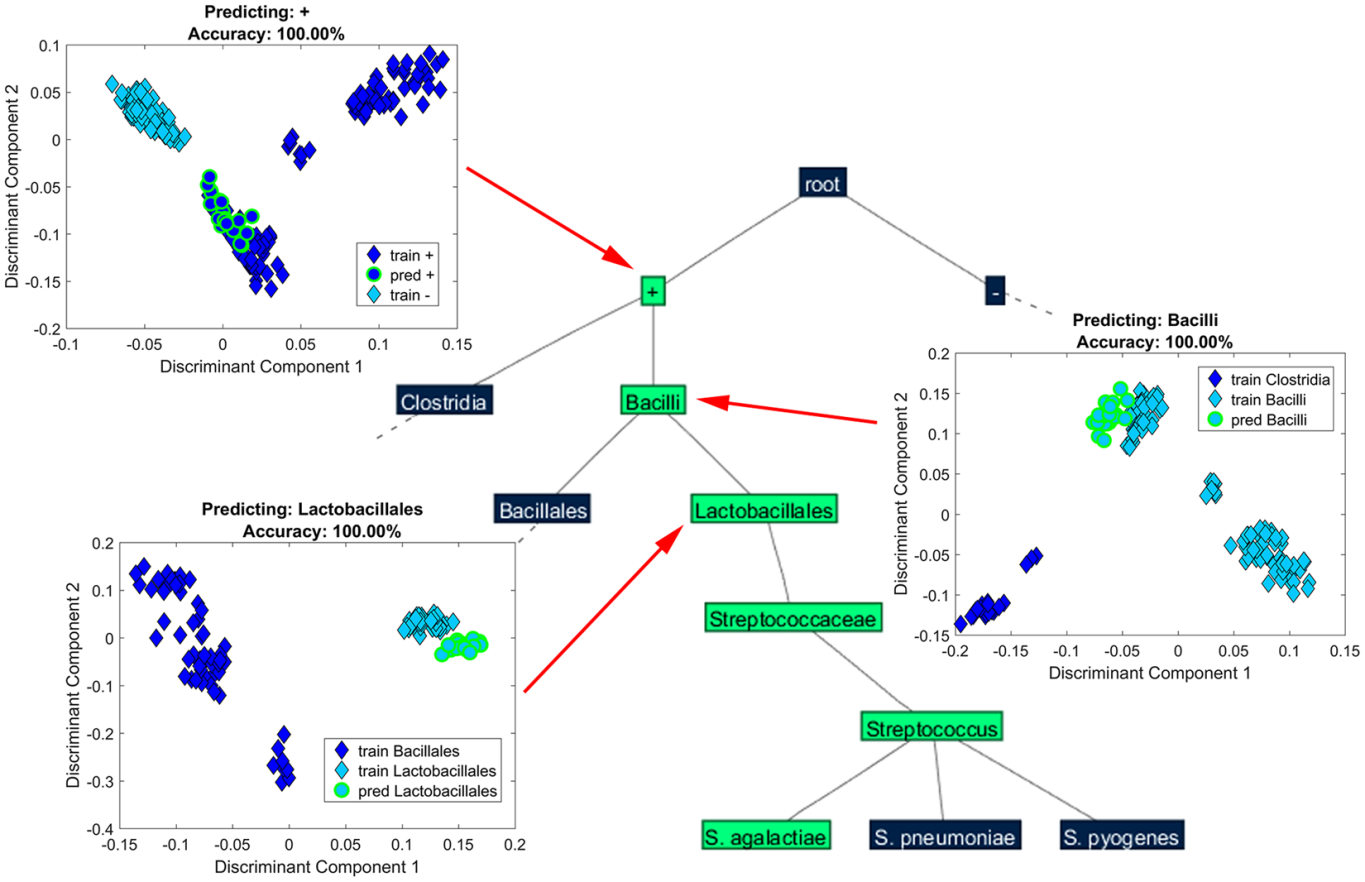
Representative leave-one-species-out scores plots for the prediction of unknown bacterial spectra. Part of the bacterial classification tree with representative discrimination plots generated for the prediction of *Streptococcus agalactiae* at various hierarchical levels using the leave-one-species-out algorithm, where *S. agalactiae* was omitted and predicted. Correctly predicted samples are indicated by a green outline. *S. agalactiae* was predicted up to genus level with 100% accuracy. The scores plotted are obtained from the ‘best’-chosen dimensionality reduction space.

Predictive capability was assessed for both bacterial and cancer high-throughput molecular data by omitting completely the class samples from the training. Prediction of bacterial species into the highest possible prediction level (i.e. a level where other species share the same class and thus parent class is not conditional upon the presence/absence of the species of interest from the dataset) was achieved for 13 out of 15 species with 100% accuracy. *Staphylococcus* spp. and *Streptococcus* spp. (Fig. 3) were predicted up to the ‘genus’ level, *Serratia* sp., *Proteus* sp., *Klebsiella* spp., *Escherichia* sp. and *Enterobacter* spp. were predicted up to the ‘family’ level, *Pseudomonas* sp. up to ‘class’ level, and the correct ‘Gram stain’ level was predicted for *Clostridium* sp. (see Supplementary Table S3).

**Figure 4:**
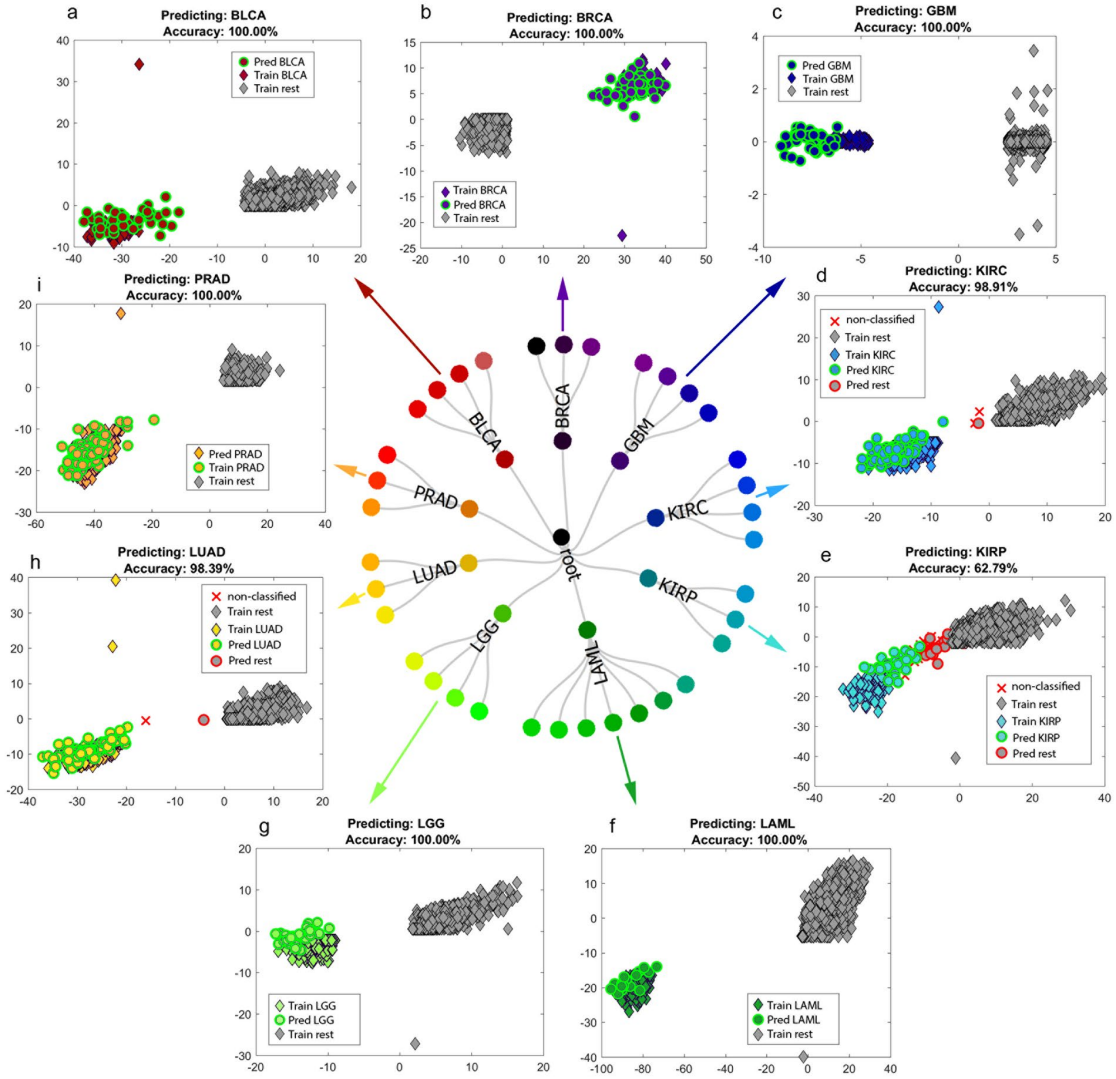
Representative leave-one-subtype-out scores plots. Discrimination plots generated for the prediction of: (**a**) ‘squamous’ subtype to bladder urothelial carcinoma (BLCA); (**b**) ‘reactive-like’ subtype to breast adenocarcinoma (BRCA); (**c**) ‘classical’ subtype to glioblastoma multiforme (GBM); (**d**) ‘ccB(2)’ subtype to kidney renal clear cell carcinoma (KIRC); (**e**) ‘PRCC Type 2’ subtype to kidney renal papillary cell carcinoma (KIRP); (**f**) ‘FAB M5’ subtype to acute myeloid leukemia (LAML); (**g**) ‘IDHmut-codel’ to lower grade glioma (LGG); (**h**) ‘proximal proliferative’ to lung adenocarcinoma (LUAD); and (**i**) ‘ETS-fusion negative’ to prostate adenocarcinoma (PRAD). Correctly predicted samples are indicated by green outline, a red ‘x’ denotes non-classified samples and misclassified samples are indicated by red outline. Axes represent discriminant components, where the second component is used only for visualization purposes. The scores plotted are obtained from the ‘best’-chosen dimensionality reduction space. Subtype assignment information is provided in the Methods and Supplementary Information Note 1. Most subtypes were correctly assigned to the respective cancer type with 95–100% accuracy (see Supplementary Table 4).

For the cancer dataset, except for one LGG subtype and one KIRP subtype, all cancer subtypes were predicted into the correct cancer type with 95–100% accuracy (Fig. 4). A list of the specific subtype prediction accuracies is provided in Supplementary Table S4.

## Discussion

We have demonstrated the effective use of a HC approach for the classification of biomolecular datasets, and extended this principle to enable robust class prediction which has potential translational utility. The application of this methodology to bacterial speciation achieves overall classification accuracies that are comparable to previously published classification methodologies that have employed mass spectrometric profiling, such as MALDI-TOF and REIMS^9,11,12^, using ‘flat’ classification approaches. Difficulties with accurately distinguishing *Enterobacteriaceae* from other species have been frequently reported in the literature, irrespective of the bacterial identification method used, owing to the high degree of biological similarity within this family. Next-generation sequencing and other emerging technologies are likely to enable refinement of bacterial taxonomic classification in the near future^16^. Nevertheless, here we are not proposing a new data acquisition method solely for improved results, but rather an alternative to the ‘flat’ classification methods that are conventionally used. With respect to performance of classification accuracy for the datasets presented herein, HC demonstrated equivalent results compared with ‘flat’ classification methods. Nonetheless, the described computational strategy can reduce classification complexity by hierarchically re-arranging data into more manageable multi-group classification problems – a strategy that is useful for hierarchical prediction which is not possible with conventional classification approaches (discussed below). Additionally, beyond what is available in the current literature^9^, here we have provided an automated solution for the selection of the ‘best’-performing statistical technique – both in terms of accuracy and computational efficiency.

Beyond HC, we demonstrate a major advantage of the proposed application that has not been exploited before – ‘hierarchical prediction’; in other words, the ability to assign upper-level taxonomic identity for classes that are not available in a given (incomplete) database. In ‘flat’ classification approaches the ‘parent-offspring’ nodal relationship is not retained between classification levels, hence hierarchical prediction is not possible with these methods. ‘Unknown’ bacterial species were identified here at the genus and family level with 100% accuracy. This approach could have significant implications in clinical microbiology where broad and narrow-spectrum antibiotics are often not species-specific, and where every hour of delay before initiating the correct antibiotic regimen can increase risk of sepsis associated mortality by 7.6%^17^. Using the proposed methodology upper hierarchy level information can be derived for rapid and more precise antibiotic treatment of unknown strains.

In contrast to HC, hierarchical clustering is an unsupervised technique used for grouping and differentiation of data according to overall similarities/differences; this can generate novel hierarchical taxonomies^18^. While this is of interest for the purpose of taxonomic reclassification, it has limited immediate clinical translational utility, pending further validatory studies. Even then, the hierarchical classification approach proposed here can easily be applied to the updated taxonomic tree.

Applying the same approach to a larger sample cohort of cancer genomic data, we found a stable 99% classification accuracy of cancer types and subsequently 74–78% accuracy at sub-type level. This enabled the probabilistic prediction of most cancer subtypes with 95–100% accuracy. With an unknown cancer subtype sample, the proposed approach thus enables the prediction of the cancer type, and subsequently suggests a subtype for it. This is especially useful for cancer types originating from the same organ of origin (e.g. KIRC and KIRP), where without any immune-histological knowledge, the model would predict the cancer type with a high degree of accuracy. Nonetheless, the pre-defined two-level cancer hierarchy used here was devised to generalize the hierarchical classification and prediction concept; showing that classes with reasonable discriminatory differences can often be identified with high accuracy, especially at upper levels. Lower prediction (and classification) accuracies are obtained in the presence of upper level misclassifications. This is a recognized limitation of a hierarchical approach where the classification error is propagated downstream.

While in the present study the HC approach was applied to bacterial mass spectrometric and cancer RNA sequencing data, the developed algorithm is not data-specific and is highly versatile. Somatic mutations, 16S rRNA, microarray gene expression, microRNA profiles and any other quantitative data can be handled. The application possibilities are diverse: (i) an unlabeled laboratory sample can be identified with various degrees of accuracy (e.g. what tissue it was obtained from, and whether it is cancerous or healthy tissue); (ii) group/taxon-specific feature selection can be retrieved, where variability or commonalities in the expression of a feature can be traced down a hierarchical tree; (iii) discovery of new species/variants/cancer subtypes if classification into existing well-classified groups is poor; and (iv) assessment of data stratification performance; where the effect of different stratification approaches on classification accuracy can be determined – to name a few.

Here we presented a general workflow for the generation of robust cross-validated models for the classification and prediction of classes at different hierarchy levels by simplifying the classification task and optimizing the statistical models used at each node. Optimizing overall performance by deriving a single global learning model for all classes is a potential alternative approach, though exploring this lies beyond the scope of the current study. While the use of a single global learning strategy sounds attractive, this may impose added complexity and introduce scalability issues.

The work presented herein has sought to demonstrate the translational utility of HC-based approaches in situations where ‘incomplete’ datasets are being evaluated, a situation that is frequently encountered in biomedical data analytics. Currently, only a tiny fraction of useable healthcare related data is actually put to translational use. This situation is unlikely to be rectified until more effective translational bioinformatics solutions are introduced capable of extracting actionable insights from the vast amounts of available data. The HC approach described here represents one such solution.

## Methods

### Data acquisition and preprocessing

Mass spectral profiles for 15 different clinically-isolated bacterial species (15–20 samples per species; cultured on Columbia horse blood agar in anaerobic conditions for *Clostridium difficile* and aerobic conditions for all other species) were acquired by rapid evaporative ionization mass spectrometry (REIMS) using an Exactive instrument (Thermo Fisher Scientific, San Jose, California)^9^. Spectral preprocessing was performed using an in-house MATLAB workflow. Spectral data were converted from RAW to mzXML format and imported at 0.001 Da resolution within the mass range of 150–2000 m/z. Noise was removed by an optimum threshold adaptively calculated using a histogram-based method^19^. Five mass spectra per sample were selected by total ion intensity (TIC) after optimum thresholding, and peak detection was performed by a 3^rd^ order derivative of a Savitzy-Golay polynomial filter^20^. Peak-matching was performed following determination of a common m/z feature vector estimated using a kernel density estimation approach^21^, where a peak with the highest peak counts was considered as a common m/z value for all the spectra. A mean mass spectrum for each bacterial species sample was derived and subsequently median-fold change normalization was applied to adjust for relative ion intensities between spectra. We have previously demonstrated the robustness of this data processing strategy^22^.

Mass spectrometric data shows heteroscedastic variation of ionic intensities, where technical variance increases as a function of signal intensity, resulting in additive or multiplicative signal. To account for the unmet assumption of multivariate statistical techniques (that noise structure is consistent throughout the whole intensity range) log variance stabilizing transformation was performed on the normalized spectra^23^.

Hierarchy structure was defined by the taxonomy of bacterial species, where: Gram staining type, class, order, family, genus and species were considered. Taxonomic information was automatically retrieved from the National Microbial Pathogen Data Resource (http://www.nmpdr.org/), and List of Prokaryotic Names with Standing in Nomenclature (http://www.bacterio.net/) online data repositories.

For the cancer genomic data, raw RNA sequencing data (RNASeqV2) for 9 cancer types (breast adenocarcinoma (BRCA), glioblastoma multiforme (GBM), kidney renal clear cell carcinoma (KIRC), kidney renal papillary carcinoma (KIRP), acute myeloid leukemia (LAML), lower grade glioma (LGG), lung adenocarcinoma (LUAD), prostate adenocarcinoma (PRAD), and bladder urothelial carcinoma (BLCA)), profiled using Illumina HiSeq, were compiled from The Cancer Genome Atlas data repository (https://tcga-data.nci.nih.gov/tcga/). Data preprocessing and filtering was performed using an in-house RNA sequencing workflow. This involved median fold change normalization followed by variance stabilizing log-2 transformation. Dubiously annotated genes were removed as well as normal tissue samples. To avoid biases, in circumstances where multiple samples were available for the same patient, a single sample was selected at random.

Cancer gene expression data was organized in a pre-defined two-level hierarchy: (i) cancer type; and subsequently (ii) cancer sub-type. Subtype assignment was based on previous literature with published gene expression unsupervised cluster assignment of mutual samples used here^24–35^. Subtypes with less than 15 samples were removed. A total of 1960 mutual samples/patients with pre-assigned subtypes were retrieved. Clusters correlated in the literature with other molecular subtypes or clinical outcomes, such as overall survival, were assigned this information label to indicate potential implications, interpretations and/or applications of the findings reported here (Supplementary Information Note 1).

### Classification algorithm

The developed algorithm is based on training a discrimination model for each node of a hierarchical tree, stratifying the data at each parent node. Starting from the upper-most node, a selective dimensionality reduction method approach^36^ is implemented using 4 different methods: (i) alternative partial least squares regression (SIMPLS)^37^, (ii) recursive linear discriminant analysis using maximum margin criterion (MMC-LDA)^38^, (iii) support vector machine (SVM) using LIBSVM version 3.20 (https://www.csie.ntu.edu.tw/~cjlin/libsvm/), and (iv) linear discriminant analysis using the Fisherfaces approach (PCA-LDA)^39^. Table 1 summarizes the eigenvalue decompositions used to derive the components in each of these.

**Table 1:** Principles of the dimensionality reduction techniques used in the classification and prediction algorithms.

Method	Method Abbrev.	Components derivation
Principal Component Analysis	PCA	Maximizes overall dataset variance without considering between-class variance
Partial Least Squares	PLS	Maximizes between-class variance without considering within-class variance
Maximum Margin Criterion	MMC	Maximizes between-class variance, while minimizing within-class variance
Linear Discriminant Analysis	LDA	Maximizes ratio of between- and within-class variation while the number of samples is greater than the number of variables
Support Vector Machines	SVM	Maximizes the margin of separation between the classes

Methods, their respective abbreviation and a descriptive derivation of their components to obtain a reduced dimensionality space. PCA and LDA were used in combination with each other or with other methods to achieve the combinatory methods: PCA-LDA and MMC-LDA.

Data are initially split into 5-fold cross-validated main test and training sets, and in turn the training set is then split into nested 5-fold cross-validated test and training sets (Supplementary Figure 1). All 4 methods outlined above were trained on the nested training set and then applied to the nested test set. The classifier achieving the highest classification accuracies throughout all the 5-fold cross-validations is saved, therefore the most generalizable and best-performing method across the data strata is selected (Supplementary Figure 1). In cases where different classifiers give equal classification accuracy, the method with the shortest computational time is chosen. This ensures that maximum performance is achieved by choosing the ‘best classifier’, whilst simultaneously ensuring computational efficiency. Via this approach, a ‘method map’ is derived which is subsequently applied to the main outer test sets, giving rise to the classification accuracies that have been reported here. In each outer cross-validation, the training and test set are fixed for the subsequent nested cross-validation in order to ensure unbiased comparison of method performance.

The user is prompted with a dialog to adjust the cross-validation rounds according to the minimum class size, ensuring robustness of the selected model. Where a parent node has only 1 down-stream node, a model is built to discriminate between the single down-stream node and other parent-related lower order nodes. For example, if a genus (level 5) has only 1 species (level 6), and the genus is related to other genera through the family (level 4), a model is built between the ‘offspring’ nodes of these related species and the single species. Samples of this single class/species which are not classified into the same species are considered as ‘non-classified’.

Probabilistic classification is performed throughout by logistic regression, where samples are assigned to the class with the highest probability. In a multi-class classification problem, classifiers are applied in a one-against-all manner^40^. In addition to nested cross-validation, classifications are repeated to obtain an average classification accuracy for each node, at each level, assessing model discriminatory performance and stability. The choice of the above linear models is determined by their scalability to high-throughput datasets and the transparency of derived discriminatory molecular signatures.

Graphic visualization is provided while the algorithm is operating to indicate workflow progress, as well as accuracies during the training phase. When complete, a set of confusion matrices are generated to summarize average classification accuracy for each node, at each taxonomic level. The quantitative accuracies given by the confusion matrices are listed in Supplementary Tables S1 and S2. In addition, we present an alternative semi-quantitative visualization technique that can intuitively summarize the performance of hierarchical classification methods, simplifying the presentation and identification of class misclassifications (Figs 1c and 2b).

### Class-prediction algorithm

Based on the HC approach, a class prediction algorithm was developed and tested using a leave-class out cross-validation strategy where each class/down-stream-node from the lower-most hierarchical level was excluded completely from the training phase, one at a time. The most efficient cross-validated method map was again determined, now based on the new cross-validated data ‘subset’. The left-out class is then applied to the method map and predicted. Starting from the root node at the upper-most hierarchical level, each sample of the left-out class is assigned to a down-stream class based on probability estimates. A probability difference between the highest probability class and the second is set as a threshold, below which samples are considered as ‘non-classified’. Percentage prediction accuracy is determined for each species after repeated predictions to assess predictive robustness.

Flowcharts illustrating the classification workflow and leave-one-out class prediction algorithms are presented in Supplementary Figure S1. Classification algorithms described here were developed in MATLAB 2014a.

### Code/data availability

The source code for the developed algorithms and data are available at:  https://bitbucket.org/iAnalytica/hierarchical-classification-publication/overview.

## Electronic supplementary material

Supplementary Information

## References

[1] Mirnezami R, Nicholson J, Darzi A (2012). Preparing for Precision Medicine. N. Engl. J. Med..

[2] Silla CNJ, Freitas AA (2010). A survey of hierarchical classification across different application domains. Data Min. and Knowl. Discov..

[3] Li J, Fong S, Zhuang Y, Khoury R (2016). Hierarchical classification in text mining for sentiment analysis of online news. IJSCAI.

[4] Cesa-Bianchi N, Gentile C, Zaniboni L (2006). Incremental Algorithms for Hierarchical Classification. J. Mach. Learn. Res..

[5] Barutcuoglu Z, Schapire RE, Troyanskaya OG (2006). Hierarchical multi-label prediction of gene function. Bioinformatics.

[6] Gupta A, Sharma VK (2015). Using the taxon-specific genes for the taxonomic classification of bacterial genomes. BMC Genomics.

[7] Balog J (2013). Intraoperative Tissue Identification Using Rapid Evaporative Ionization Mass Spectrometry. Sci. Transl. Med..

[8] Hutsebaut D (2006). Raman microspectrometry as an identification tool within the phylogenetically homogeneous ‘Bacillus subtilis’-group. Sys. Appl. Microbiol..

[9] Strittmatter N (2014). Characterization and identification of clinically relevant microorganisms using rapid evaporative ionization mass spectrometry. Anal. Chem..

[10] Anhalt JP, Fenselau C (1975). Identification of bacteria using mass spectrometry. Anal. Chem..

[11] Richter SS (2013). Identification of Enterobacteriaceae by matrix-assisted laser desorption/ionization time-of-flight mass spectrometry using the VITEK MS system. Eur. J. Clin. Microbiol. Infec. Dis..

[12] Sauer S, Kliem M (2010). Mass spectrometry tools for the classification and identification of bacteria. Nature Rev. Microbiol..

[13] Bizzini A, Greub G (2010). Matrix-assisted laser desorption ionization time-of-flight mass spectrometry, a revolution in clinical microbial identification. Clin. Microbiol. Infect..

[14] Cherkaoui A (2010). Comparison of Two Matrix-Assisted Laser Desorption Ionization-Time of Flight Mass Spectrometry Methods with Conventional Phenotypic Identification for Routine Identification of Bacteria to the Species Level. J. Clin. Microbiol..

[15] Martiny D (2012). Comparison of the Microflex LT and Vitek MS systems for routine identification of bacteria by matrix-assisted laser desorption ionization-time of flight mass spectrometry. J. Clin. Microbiol..

[16] Baylis, C., Uyttendaele, M., Joosten, H. & Davies, A. The Enterobacteriaceae and their significance to the food industry. *ILSI Europe* 1–48 (2011).

[17] Kumar A (2006). Duration of hypotension before initiation of effective antimicrobial therapy is the critical determinant of survival in human septic shock. Crit. Care Med..

[18] Slabbinck B, Waegeman W, Dawyndt P, De Vos P, De Baets B (2010). From learning taxonomies to phylogenetic learning: Integration of 16S rRNA gene data into FAME-based bacterial classification. BMC Bioinform..

[19] Otsu N (1975). A threshold selection method from Gray-level histograms. IEEE Trans. Syst., Man, Cybern., Syst..

[20] Savitzky AGMJE (1964). Smoothing and differentiation of data by simplified least squares procedures. Anal. Chem..

[21] Fushiki T, Fujisawa H, Eguchi S (2006). Identification of biomarkers from mass spectrometry data using a “common” peak approach. BMC Bioinform..

[22] Veselkov KA (2011). Optimized preprocessing of ultra-performance liquid chromatogrpahy/mass spectrometry urinary metabolic profiles for improved information recovery. Anal. Chem..

[23] Veselkov KA (2014). Chemo-informatic strategy for imaging mass spectrometry-based hyperspectral profiling of lipid signatures in colorectal cancer. Proc. Natl. Acad. Sci. USA.

[24] Network TCGAR (2013). Comprehensive molecular characterization of clear cell renal cell carcinoma. Nature.

[25] Network TCGAR (2016). Comprehensive molecular characterization of papillary renal-cell carcinoma. N. Eng. J. Med..

[26] Network TCGAR (2014). Comprehensive molecular characterization of urothelial bladder carcinoma. Nature.

[27] Network TCGAR (2015). Comprehensive molecular portraits of invasive lobular breast cancer. Cell.

[28] Network TCGAR (2014). Comprehensive molecular profiling of lung adenocarcinoma. Nature.

[29] Network TCGAR (2015). Comprehensive, integrative genomic analysis of diffuse lower-grade gliomas. N. Engl. J. Med..

[30] Network TCGAR (2013). Genomic and epigenomic landscapes of adult de novo acute myeloid leukemia. N. Engl. J. Med..

[31] Network TCGAR (2015). The molecular taxonomy of primary prostate cancer. Cell.

[32] Network TCGAR (2013). The somatic genomic landscape of glioblastoma. Cell.

[33] Brannon AR (2010). Molecular stratification of clear cell renal cell carcinoma by consensus clustering reveals distinct subtypes and survival patterns. Genes Cancer.

[34] Bennett JM (1976). Proposals for the classification of the acute leukaemias French-American-British (FAB) co-operative group. Br. J. Haematol..

[35] Verhaak RG (2010). Integrated genomic analysis identifies clinically relevant subtypes of glioblastoma characterized by abnormalities in PDGFRA, IDH1, EGFR, and NF1. Cancer Cell.

[36] Secker AD (2007). An experimental comparison of classification algorithms for the hierarchical prediction of protein function. Expert Update.

[37] De Jong S (1993). SIMPLS: An alternative approach to partial least squares regression. Chemometr. Intell. Lab.

[38] Li H, Jiang T, Zhang K (2004). Efficient and robust feature extraction by maximum margin criterion. IEEE Trans. Neural Netw..

[39] Belhumeur PN, Hespanha JP, Kriegman DJ (1997). Eigenfaces vs. Fisherfaces: Recognition using class specific linear projection. IEEE Trans. Pattern Anal. Mach. Intell..

[40] Lorena, A. C., Carvalhom A. C. P. L. F. & Gama, J. M. A review on the combination of binary classifiers in multiclass problems. *Artif. Intell. Rev*. **30**, 10.1007/s10462-009-9114-9 (2008).

