# Supplementary material for: Translational utility of a hierarchical classification strategy in biomolecular data analytics
Source: Sci Rep. 2017 Nov 3;7:14981. doi: 10.1038/s41598-017-14092-7 (PMC5670129; doi:10.1038/s41598-017-14092-7)
Supplement: Supplementary file 1 — Supplementary Information [file 41598_2017_14092_MOESM1_ESM.doc]

**Translational utility of a hierarchical classification strategy in biomolecular data analytics**

Dieter Galea^1^, Paolo Inglese^1^, Lidia Cammack^1^, Nicole Strittmatter^1^, Monica Rebec^1^, Reza Mirnezami^1^, Ivan Laponogov^1^, James Kinross^1^, Jeremy Nicholson^1^, Zoltan Takats^1^ & Kirill A. Veselkov^1*^

^1^Computational and Systems Medicine, Department of Surgery and Cancer, Faculty of Medicine, Imperial College London, London, United Kingdom. ^*^Correspondence should be addressed to K.A.V (kirill.veselkov04@imperial.ac.uk).

SUPPLEMENTARY INFORMATION

**Supplementary Figure 1 – Flow Charts**

**
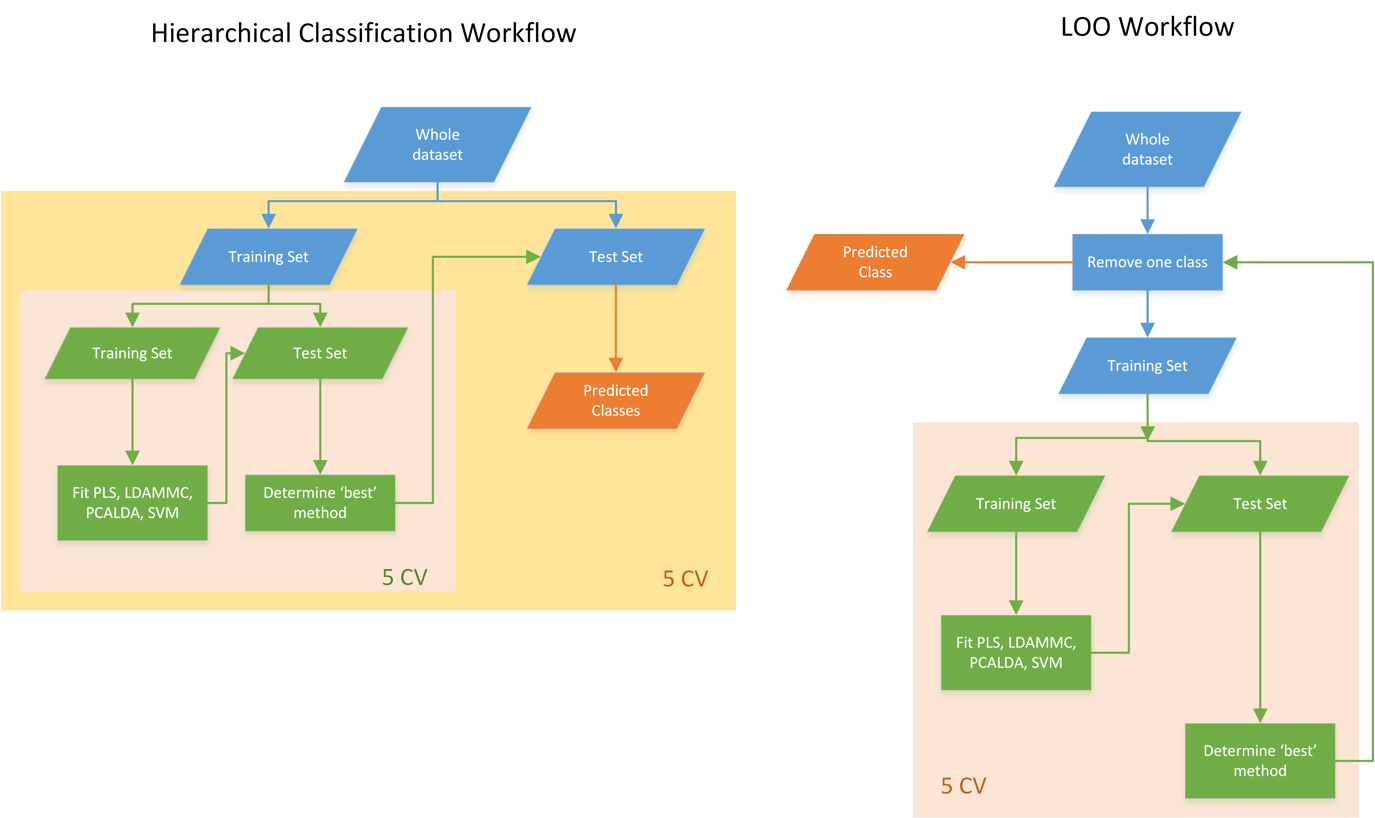
**

A

B

**Supplementary Figure 1. Flow charts for the classification and class prediction algorithms**. A) Nested cross-validated selective classifier approach algorithm indicating splitting and fixing of training/test set, dimensionality reduction using MMCLDA, SVM, PCALDA and PLS, classification and prediction by logistic regression and the most accurate method is applied to the test set for classification of the test set. B) Modification to the probabilistic classification model for leave-one-out (LOO) prediction where the best model is used to classify a class that is not part of the model training phase. Predictions are given for each hierarchical level.

**Supplementary Figure 2 - Model plots for bacterial species differentiation**

**
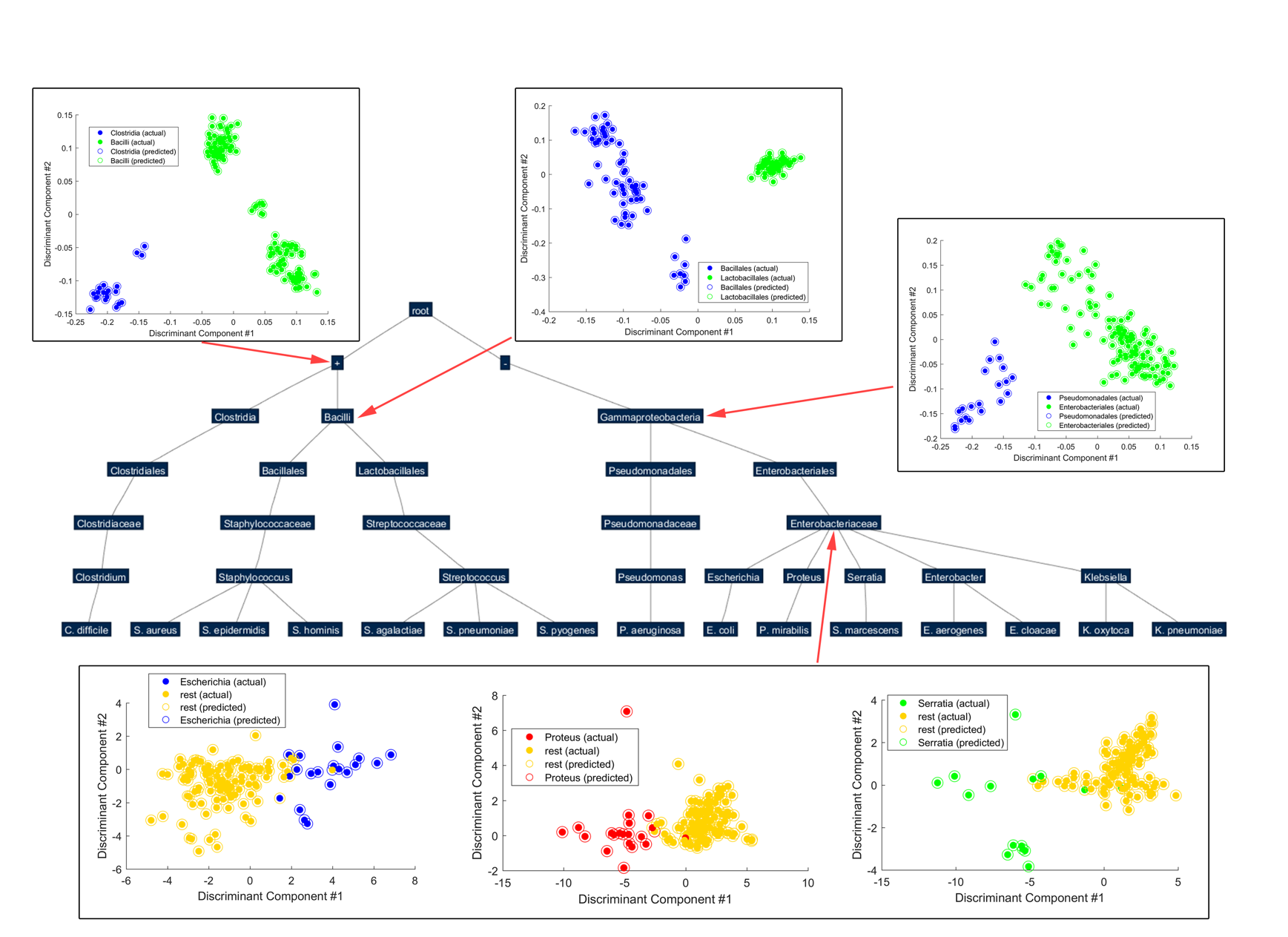
**

A

B

C

D

**Supplementary Figure 2. Discrimination plots for the classification of bacterial species at different nodes and levels.** Score plots for the first two components in the ‘best’-selected reduced space representing classification of: A) Gram-positive bacteria into Class Clostridia or Bacilli; B) Bacilli samples into Order Bacillales or Lactobacillales; C) Gammaproteobacteria samples into Pseudomonadales and Enterobacteriales; and D) Enterobacteriaceae into the genera Escherichia, Proteus and Serratia. Solid markers indicate the actual classes, while outlines indicate the predicted class. Misclassifications are indicated by colors mismatch of the solid marker and outline. In a multi-class problem, as in D), a model was built for each class in a one-vs-rest approach; with the aggregated class represented as ‘rest in the plots.

**Supplementary Table 1**. **Classification accuracies for the different bacterial species**. Classification accuracy for each bacterial species represented semi-quantitatively in Figure 1.

| **Species** | **Classification Accuracy (%)** |
| --- | --- |
| *Clostridium difficile* | 100 |
| *Staphylococcus aureus* | 99 |
| *Staphylococcus epidermidis* | 75 |
| *Staphylococcus hominis* | 84 |
| *Streptococcus agalactiae* | 100 |
| *Streptococcus pneumoniae* | 94 |
| *Streptococcus pyogenes* | 95 |
| *Pseudomonas aeruginosa* | 100 |
| *Escherichia coli* | 69 |
| *Proteus mirabilis* | 80 |
| *Serratia marcescens* | 85 |
| *Enterobacter aerogenes* | 55 |
| *Enterobacter cloacae* | 79 |
| *Klebsiella oxytoca* | 89 |
| *Klebsiella pneumoniae* | 66 |

**Supplementary Table 2**. **Classification accuracies for the different cancer subtypes**. Classification accuracy for each cancer subtypes and their respective cancer type represented semi-quantitatively in Figure 2.

| **Cancer Type** | **Cancer Subtype** | **Classification Accuracy (%)** |
| --- | --- | --- |
| BRCA | Reactive-like | 79 |
|  | Immune-related | 81 |
|  | Proliferative | 51 |
| GBM | Classical | 67 |
|  | Mesenchymal | 75 |
|  | Neural | 35 |
|  | Proneural | 65 |
| KIRC | ccB(1) | 70 |
|  | ccA | 90 |
|  | ccB(2) | 58 |
|  | ccA/ccB | 48 |
| KIRP | PRCC Type 1 | 61 |
|  | PRCC Type 1(2) | 92 |
|  | PRCC Type 2 | 82 |
| LAML | FAB M5 | 56 |
|  | FAB M3 | 85 |
|  | Poorest survival | 33 |
|  | Low survival (1) | 76 |
|  | FAB M4 | 80 |
|  | Low survival (2) | 68 |
|  | FAB M1 | 72 |
| LGG | IDHmut-non-codel | 93 |
|  | IDHwt/IDHmut-non-codel | 95 |
|  | IDHmut-codel | 97 |
|  | heterogeneous | 97 |
| LUAD | TRU | 89 |
|  | Proximal Inflammatory | 80 |
|  | Proximal Proliferative | 85 |
| PRAD | ETS-fusion +ve(1) | 87 |
|  | ETS-fusion +ve(2) | 88 |
|  | ETS-fusion –ve | 85 |
| BLCA | Low KRT/EGFR | 82 |
|  | High FGFR3 mut/amp/fus | 80 |
|  | Squamous | 84 |
|  | Low FGFR3 mRNA | 67 |

**Supplementary Table 3. Predicted levels for different bacterial species.** Hierarchical level at which bacterial species were predicted with 100% prediction accuracy. Lower levels could not be predicted for *Clostridium difficile*, *Escherichia coli, Proteus mirabilis, Pseudomonas aeruginosa,* and *Serratia marcescens* since lower levels were not shared by other species and hence were excluded upon omission of the species from the dataset. *Enterobacter* spp. and *Klebsiella* spp. were poorly predicted at the genus level.

| Species | Predicted level |
| --- | --- |
| *Clostridium difficile* | Gram |
| *Enterobacter aerogenes* | Family |
| *Enterobacter cloacae* | Family |
| *Escherichia coli* | Family |
| *Klebsiella oxytoca* | Family |
| *Klebsiella pneumoniae* | Family |
| *Proteus mirabilis* | Family |
| *Pseudomonas aeruginosa* | Class |
| *Serratia marcescens* | Family |
| *Staphylococcus aureus* | Genus |
| *Staphylococcus epidermidis* | Genus |
| *Staphylococcus hominis* | Genus |
| *Streptococcus agalactiae* | Genus |
| *Streptococcus pneumoniae* | Genus |
| *Streptococcus pyogenes* | Genus |

**Supplementary Table 4. Cancer sub-type prediction accuracies.** Average cancer type prediction accuracies for each cancer sub-type. Details for the derivation of sub-types are given in Supplementary Note 1.

| Sub-type | Predicted Cancer Type | Prediction Accuracy (%) |
| --- | --- | --- |
| Reactive-like | BRCA | 100 |
| Immune-related |  | 100 |
| Proliferative |  | 96 |
| Classical | GBM | 100 |
| Mesenchymal |  | 100 |
| Neural |  | 100 |
| Proneural |  | 100 |
| ccB(1) | KIRC | 99 |
| ccA |  | 97 |
| ccB(2) |  | 99 |
| ccA/ccB |  | 95 |
| PRCC Type 1 | KIRP | 100 |
| PRCC Type 1(2) |  | 100 |
| PRCC Type 2 |  | 63 |
| FAB M5 | LAML | 100 |
| FAB M3 |  | 100 |
| Poorest Survival |  | 100 |
| Low Survival(1) |  | 100 |
| FAB M4 |  | 100 |
| Low Survival(2) |  | 100 |
| FAB M1 |  | 100 |
| IDHmut-non-codel | LGG | 100 |
| IDHwt/IDHmut-non-codel |  | 52 |
| IDHmut-codel |  | 100 |
| Heterogenous |  | 100 |
| TRU | LUAD | 100 |
| Prox. Inflammatory |  | 97 |
| Prox. Proliferative |  | 98 |
| ETS-fusion –ve | PRAD | 100 |
| ETS-fusion +ve(2) |  | 100 |
| ETS-fusion +ve(1) |  | 100 |
| Low FGFR3 mRNA | BLCA | 100 |
| Squamous |  | 100 |
| High FGFR3 mut/amp/fus |  | 100 |
| Low KRT/EGFR |  | 100 |

**Supplementary Note 1 – Cancer subtype information retrieved from literature**

**Kidney Renal Clear Cell Carcinoma – KIRC**

KIRC mRNA clusters were reported^24^ to relate to the clear cell type A (ccA) and clear cell type B (ccB) expression subtypes^28^, with cluster 1 found to be correlated to ccA, cluster 2 and 3 correlated with ccB, and cluster 4, while not correlated with neither ccA nor ccB, is suggested to account for ~15% tumors previously unclassified in ccA/ccB classes.

**Kidney Renal Papillary Cell Carcinoma – KIRP**

KIRP mRNA clusters 1 and 3 were reported^25^ to be dominated by papillary renal cell carcinoma (pRCC) Type I, Stage I-II while cluster 2 was dominated by pRCC Type II, Stage III-IV.

**Acute Myeloid Leukemia – LAML**

LAML mRNA clusters were reported^30^ to correlate with the French-American-British (FAB) subtype classification of acute leukemias^39^, with cluster 3 represented FAB subtype M3 (acute promyelocytic leukemia), cluster 4 associated with FAB subtype M1 (AML with minimal maturation), cluster 5 with FAB subtype M4 (acute myelomonocytic leukemia), and cluster 7 with FAB subtype M5 (acute monoblastic or monocytic leukemia). Clusters 1, 2, and 6 were not correlated with FAB subtypes and thus survival information was retrieved from the Kaplan Meier overall survival curve provided in the original study.

**Lower Grade Glioma – LGG**

The Cancer Genome Atlas Research Network^29^ report 4 mRNA clusters for LGG. Cluster 2 was enriched for IDHwt (Isocitrate dehydrogenase wild-type) tumor samples with the worst overall survival compared to the other clusters, cluster 1 was correlated with DNA methylation subtype M5 and DNA methylation subtype M3 identified in the same study, cluster 3 was found to be composed entirely of IDH-mut-codel (IDH-mutation 1p/19q codeletion) gliomas, while cluster 4 was found not to be associated with any specific subtype.

**Prostate Adenocarcinoma – PRAD**

3 mRNA clusters were found to be highly correlated with ETS (E26 transformation-specific transcription factors) fusion status by The Cancer Genome Atlas Research Network^31^, with cluster 1 consisted primarily of ETS-negative tumors while clusters 2 and 3 reported to contain ETS fusion-positive tumors.

**Glioblastoma Multiforme – GBM**

Proneural, neural, classical and mesenchymal molecular subclasses of GBM are transcriptomic clinically-relevant subtypes reported in literature^40^. Cluster assignment for specific samples were retrieved from The Cancer Genome Atlas Research Network^32^.

**Breast Adenocarcinoma – BRCA**

The Cancer Genome Atlas Research Network^27^ reported 3 breast tumor types based on mRNA sequencing data; reactive-like, proliferative, and immune-related. These were identified to have several genomic differences at the mRNA and protein/phosphoprotein level but not somatic mutations or DNA copy-number alterations.

**Bladder Urothelial Carcinoma – BLCA**

4 main mRNA subtypes were identified for BLCA in the study by The Cancer Genome Atlas Research Network^26^. Cluster 1 samples were enriched with FGFR3 alterations, FGFR3 expression and reduced FGFR3-related miRNA expression. Cluster 3 samples were dominated by squamous features, mRNA subtype 4 showed low expression of FGFR3 mRNA, while cluster 2 showed lower KRT and EGFR expression compared to the other subtypes.

**Lung Adenocarcinoma – LUAD**

The Cancer Genome Atlas Research Network^28^ propose 3 molecular subtypes for LUAD, based on the clustering of mRNA. Namely: Terminal Respiratory Unit (TRU), proximal-inflammatory and proximal-proliferative transcriptional subtypes. Proximal-proliferative was found to be enriched for KRAS mutations, inactivation of STK11 tumor suppressor gene, and reduced gene expression. Proximal-inflammatory was associated with the co-mutation of NF1 and TP53, while TRU cluster samples were highly EGFR-mutated and expressing kinase fusion.
